# The Impact of Comorbid Depression on Cognitive Reserve in Patients with Alzheimer’s Disease: A Case‐Control Study

**DOI:** 10.1002/alz70861_108988

**Published:** 2025-12-23

**Authors:** Arwa Ayman Nagah Ahmed Shahin, Mohamed Khaled Ali Mohamed Ali Zid, Mohamed Hosni Kamel Elkellawi

**Affiliations:** ^1^ Cairo University Faculty of Medicine, Cairo, Cairo Egypt

## Abstract

**Background:**

The cognitive reserve (CR) enables individuals to maintain cognitive function despite neurodegenerative pathology. Depression is a frequent comorbidity in Alzheimer’s disease (AD), and growing evidence suggests it may negatively affect CR by limiting neural compensation and reducing engagement in cognitively enriching activities. This study investigates whether comorbid depression in patients with Alzheimer’s disease is associated with diminished cognitive reserve.

**Method:**

This case‐control study involved 90 patients with clinically diagnosed mild‐to‐moderate AD. AD cases (n = 45) were comorbid for major depressive disorder (AD+Dep), while controls (n = 45) were AD but without depression. Cognitive reserve was estimated by a reliable composite score derived from educational attainment, premorbid IQ (as measured by the National Adult Reading Test), and occupational complexity. Depression was diagnosed using DSM‐5 criteria and assessed using the Geriatric Depression Scale (GDS). Statistical comparisons were done using t‐tests and regression models adjusted for age and severity of disease.

**Result:**

The AD+Dep group's cognitive reserve scores were significantly lower (mean CR index = 5.8 ± 1.1) compared to the AD‐only group (6.7 ± 0.9; *p* < 0.001). This correlation stayed significant even after controlling for age, sex, and MMSE scores. Higher GDS scores were negatively correlated with CR index (r = –0.41, *p* < 0.01), suggesting that higher depressive severity was related to declining reserve.

**Conclusion:**

The depression in AD patients is associated with reduced cognitive reserve, which may increase vulnerability to more accelerated cognitive decline. These findings suggest that intervention of depressive symptoms may be implicated in the preservation of cognitive resilience in AD.

